# Parametric Power Spectral Density Analysis of Noise from Instrumentation in MALDI TOF Mass Spectrometry

**Published:** 2007-09-17

**Authors:** Hyunjin Shin, Miray Mutlu, John M. Koomen, Mia K. Markey

**Affiliations:** 1 Department of Biostatistics and Computational Biology, Dana-Farber Cancer Institute, Boston, MA, U.S.A; 2 Department of Electrical and Electronics Engineering, Bilkent University, Bilkent Ankara, Turkey; 3 H. Lee Moffitt Cancer Center and Research Institute, Tampa, FL, U.S.A; 4 The University of Texas Department of Biomedical Engineering, Austin, TX, U.S.A

**Keywords:** Mass, Spectrometry, Mass, Matrix-Assisted Laser Desorption-Ionization, Noise, Artifacts, Fourier Analysis, Signal Processing, Computer-Assisted, Computer Simulation, Models, Computer

## Abstract

Noise in mass spectrometry can interfere with identification of the biochemical substances in the sample. For example, the electric motors and circuits inside the mass spectrometer or in nearby equipment generate random noise that may distort the true shape of mass spectra. This paper presents a stochastic signal processing approach to analyzing noise from electrical noise sources (i.e., noise from instrumentation) in MALDI TOF mass spectrometry. Noise from instrumentation was hypothesized to be a mixture of thermal noise, 1/f noise, and electric or magnetic interference in the instrument. Parametric power spectral density estimation was conducted to derive the power distribution of noise from instrumentation with respect to frequencies. As expected, the experimental results show that noise from instrumentation contains 1/f noise and prominent periodic components in addition to thermal noise. These periodic components imply that the mass spectrometers used in this study may not be completely shielded from the internal or external electrical noise sources. However, according to a simulation study of human plasma mass spectra, noise from instrumentation does not seem to affect mass spectra significantly. In conclusion, analysis of noise from instrumentation using stochastic signal processing here provides an intuitive perspective on how to quantify noise in mass spectrometry through spectral modeling.

## Introduction

In recent years, matrix-assisted laser desorption/ionization time-of-flight mass spectrometry (MALDI TOF MS) and its variants (e.g. surface-enhanced laser desorption/ionization time-of-flight MS) analyzed with computational pattern recognition algorithms have attracted attention as tools for early diagnosis of cancer. The key role of MALDI TOF or SELDI TOF MS for early cancer diagnosis is to identify differences due to pathological changes between the mass spectra of diseased samples and those of controls so that pattern recognition algorithms can learn statistically dissimilar patterns. However, because most such pattern differences in mass spectra of samples such as plasma/serum are very subtle, noise can cause false positives or false negatives in peak detection by distorting the true shape of the mass spectrum. Thus, several studies have investigated methods for characterizing or reducing noise in order to improve the sensitivity of MS (Coombes et al. 2005; Baggerly et al. 2003; Lee et al. 2003; Liu et al. 2003; Satten et al. 2004; Wagner et al. 2003; Zhu et al. 2003b; Malyarenko et al. 2005; Keller and Li, 2000; Krutchinsky and Chait, 2002; Shin et al. 2004; Anderle et al. 2004; Coombes et al. 2003; Neville et al. 2003; Wang et al. 2003; Zhu et al. 2003a; Qu et al. 2003; Andreev et al. 2003; Statheropoulos et al. 1999; Hastings et al. 2002).

To date, most efforts for noise reduction, particularly in MALDI TOF MS, have focused on eliminating the baseline and reducing high frequency noise (Coombes et al. 2005; Baggerly et al. 2003; Lee et al. 2003; Liu et al. 2003; Satten et al. 2004; Wagner et al. 2003; Zhu et al. 2003b; Malyarenko et al. 2005; Statheropoulos et al. 1999; Hastings et al. 2002). The baseline is a monotonically decreasing bias in the mass spectrum that originates from matrix clusters formed during the ionizing process. To eliminate this baseline, it is heuristically estimated (Baggerly et al. 2003; Coombes et al. 2003; Liu et al. 2003; Neville et al. 2003; Wagner et al. 2003) and then subtracted from the original mass spectrum. For the baseline estimate, a local average or minimum intensity within a moving window (Baggerly et al. 2003), the piecewise linear regression line (Wagner et al. 2003; Neville et al. 2003), or the convex hull of the intensities (Liu et al. 2003) have often been used. On the other hand, high frequency noise appears in the mass spectrum as fast varying ripples or irregular peaks at certain m/z points. A number of factors such as electrical interference, random ion motions, statistical fluctuation in the detector gain, or chemical impurities may be involved with the occurrence of the high frequency noise. Heuristic approaches have been predominantly used to reduce high frequency noise. For example, moving averaging filters (Liu et al. 2003), Gaussian kernel filters (Wang et al. 2003; Zhu et al. 2003b), principal component analysis (PCA) (Statheropoulos et al. 1999) and the wavelet transform (WT) (Coombes et al. 2005; Zhu et al. 2003a; Qu et al. 2003) are the common techniques for high frequency noise reduction. Andreev et al. obtained power spectral density estimates of the high frequency noise through nonparametric power spectral density estimation and designed a matched filter to reduce the noise adaptively (Andreev et al. 2003). Most manufacturers also provide noise reduction algorithms such as a moving average filter in their products; however, it is difficult to obtain optimal filtering results because the users must determine the filter parameters iteratively through experimentation or based on previous experience.

The noise reduction approaches introduced above have been established based on empirical insight rather than on rigorous statistical noise analysis; therefore, the parameters of these algorithms have been determined in an *ad hoc* manner. Few studies have investigated the noise sources in MS and attempted to model the noise by measuring its statistical characteristics. Anderle et al attempted to represent the noise magnitude variance in liquid chromatographic MS (LC MS) as a combination of quadratic and linear models (Anderle et al. 2004). Similarly, Hastings et al fitted the log transformed noise level to a sum of two normal distributions, and compared the performance of the average and median filters based on their noise model (Hastings et al. 2002). However, since these studies have been done mainly using statistical error analysis rather than stochastic signal processing, they cannot provide sufficient perspective on how noise varies with time and frequency. Malyarenko et al. developed a numerical baseline model using the phenomenon of exponentially decaying charge accumulation on the ion detector (Malyarenko et al. 2005). Shin et al. also proposed a noise model for MALDI TOF MS, where we categorized noise into three types: noise from instrumentation, noise from random ion motions and statistical fluctuations in the ion detector, and chemical noise. Then, we hypothesized that the observed noise is a result of multiplication and addition of these hidden components. Additionally, we reported the results of non-parametric power spectral density analysis on noise from instrumentation (Shin et al. 2004). Similar efforts to reduce chemical noise were also made by some manufacturers. For example, Applied Biosystems Inc. developed an algorithm based on the Fourier transform and notch filtering to minimize the effect of chemical impurities on mass spectra (Baranov, 2001). They tried to identify periodic patterns of chemical noise in mass spectra using the Fourier transform, and to reduce signal deterioration by eliminating these periodic patterns using a notch filter. However, their approach does not seem to be strictly model-based in the sense that they did not build a model for chemical noise from the frequency representation.

These model-based studies represent an important advance over heuristic approaches. The lack of knowledge on statistical characteristics of the signal and noise in heuristic approaches may lead to the design of noise reduction algorithms or digital filters that deteriorate the true signal rather than restore it. However, more work needs to be done towards complete noise characterization. Prior studies may have oversimplified the noise sources or disregarded the importance of power spectral density analysis. For example, most noise analyses have not explicitly distinguished the subtypes of the high frequency noise; however, various electrical, physical, and chemical components of the mass spectrometer may generate subtypes of noise with different characteristics. Therefore, in order to elucidate the stochastic characteristics of noise in mass spectrometry, such individual noise components must be carefully separated and analyzed. In addition to noise subtype isolation and measurement, power spectral density estimation is also critical in noise characterization because this method can provide guidance for digital filter design by showing the power distribution of noise over frequencies, which determines the magnitude and period of signal fluctuation due to noise in the mass spectrum.

As part of our effort for modeling noise in MALDI TOF mass spectrometry, we describe a method in which we have isolated noise from instrumentation occurring in the MALDI TOF mass spectrometer and obtained the signal model for this type of noise using parametric power spectral density estimation. By “noise from instrumentation”, we mean the interference caused by electrical sources inside or near the mass spectrometer including thermal noise from the transimpedance amplifier, power supply and power line noise, and electrical interference from the ion accelerator pulse. In following sections, we introduce the fundamental theory of random signal modeling based on parametric power spectral density estimation and our approaches to investigating the spectral characteristics of noise from instrumentation in MALDI TOF mass spectrometry.

## Fundamental Theory

In general, a random process does not show regular patterns in the time domain like a sine wave because many signals of different frequencies and phases are added together. The power spectral density of a random process provides the power distribution of the signal with respect to frequencies. If there is a high value at a certain frequency in a power spectral density, the corresponding random process has a strong sine wave with that frequency in the time domain (Proakis and Manolakis, 2000). The simplest way of estimating the power spectral density of a random process is to calculate the absolute square of the Fourier transform of a given realization, which is referred to as the periodogram. Power spectral density estimation methods based on the periodogram are called nonparametric methods because these methods derive a power spectral density estimate from given realizations without any background information on the data source. However, nonparametric methods suffer from poor frequency resolution and spectral leakage effects due to the finite length of data. The lack of resolution in nonparametric estimation becomes more problematic when the sampling frequency is very high but the data length is relatively short. In this case, a non-parametric power spectral density estimate would provide power information on only a relatively small number of frequencies within a wide range of frequencies (Proakis and Manolakis, 2000). Spectral leakage causes ripples in a power spectral density estimate, which makes it difficult to identify true periodic components in the signal.

Parametric power spectral density analysis can overcome these drawbacks by estimating the parameters of a linear system under the assumption that the observed random signal is the output of the linear model when a random signal with a white frequency spectrum is given as input. Once a model is established, a high-resolution power spectral density estimate free from spectral leakage can be obtained since the power spectral density of the random signal is determined by the parameters of the linear system (Proakis and Manolakis, 2000). Table 1 briefly summarizes the advantages and disadvantages of non-parametric and parametric power spectral density estimation.

In parametric power spectral density estimation, the difference equation between the input random signal and the observed signal in the time domain can be written as:

(1) $$\begin{array}{l} x\left(n\right)+a_{1}x\left(n-1\right)+\cdots +a_{p}x\left(n-p\right)\\ =w\left(n\right)+b_{1}w\left(n-1\right)+\cdots +b_{q}\left(n-q\right) \end{array}$$

In the above equation, *x*(*n*) denotes the observed signal system at the *n*th time index, and *w*(*n*), the input random signal at the same time index. *H*(*f*), the Fourier representation of the linear system, is defined as the ratio of *X*(*f*) and *W*(*f*), the Fourier representations of *x*(*n*) and *w*(*n*) and it is uniquely determined by *a*_1_, …, *a_p_* and *b*_1_, …, *b_q_*. The power spectral density of the random signal *S_X_*(*f*) is obtained using the following equation:

(2) $$S_{X}\left(f\right)={\left|H\left(f\right)\right|}^{2}S_{w}\left(f\right)$$

where *S_w_*(*f*) is the power spectral density of the input signal with a white spectrum (Proakis and Manolakis, 2000).

Three different types of random processes can be generated using the linear model. When *b*_1_, …, *b_q_* *=* 0, the process produced by the linear model is called an autoregressive (AR) process of order *P*. When *a*_1_, …, *a_p_* *=* 0, the resulting process is called a moving average (MA) process of order *q*. Otherwise, the process is called an autoregressive-moving average (ARMA) process of order *p* and *q*. Generally, these three models could be exchanged if models of infinite order be allowed. However, among these three types, the AR model is most commonly used for power spectral density estimation because it can show narrow frequency components more accurately than the others with simple linear equations for parameter estimation (Proakis and Manolakis, 2000).

The Burg algorithm estimates the power spectral density using an AR model. The AR parameters are estimated by minimizing the forward and backward residuals of the model, which are defined as the error between the given random signal and their corresponding estimators at *n* and *n* – *p* (Proakis and Manolakis, 2000). In general, power spectral density estimates obtained by the Burg algorithm have high frequency resolution (Proakis and Manolakis, 2000), and are more unbiased and stable than other power spectral density estimation algorithms using an AR model such as the Yule-Walker algorithm and least square estimator (de Waele and Broersen, 2000).

Ideally, an infinite measurement of a random process is desired to develop a most accurate model; however, in reality, measurements have finite length due to practical limitations of instrumentation. For example, in MALDI TOF mass spectrometry, the maximum signal length is determined by the instrument according to a pre-defined limit on the maximum mass to charge ratio. In recognition of this common problem, de Waele and Broersen extended the Burg algorithm to obtain a more accurate model using multiple segments from a random process than can be achieved using a single realization of the process (de Waele and Broersen, 2000). Like the Burg algorithm, this algorithm also estimates the model parameters by minimizing the forward and backward residuals; however, the revised algorithm attempts to minimize residuals from multiple segments simultaneously (de Waele and Broersen, 2000). This extended Burg algorithm generates a more accurate model than parameter averaging methods, which develop a final model by averaging the parameters of the models derived from individual segments (de Waele and Broersen, 2000).

The model order must be carefully determined so that the model can represent the given segments well, while avoiding overfitting. In general, the residuals decrease as the model order increases, so the modeling process must be stopped at some point even though the residuals are still decreasing. In the Burg algorithm, the Akaike’s information criterion (AIC) is employed to select the optimum model order (de Waele and Broersen, 2000). The AIC is represented as the sum of the model order and the log residual of the model with respect to the given random process. The parameter estimation of the Burg algorithm stops when the AIC is minimized. When errors between the estimated model and true random process is normally distributed, the AIC is defined as the following equation

(3) $$\begin{array}{l} AIC\left(p\right)=\text{ln}\left(RES\left(p\right)\right)+\frac{2p}{N} \end{array}$$

where *RES*(*p*) is the residual variance of the model of order *p*, and *N* is the length of a given signal realization (de Waele and Broersen, 2000; Akaike, 1974). In the Burg algorithm for multiple segments, the above definition of AIC is slightly modified so that it may reflect the fact that the variance of the estimated parameters becomes lower than when a single segment is used by a factor of *S*, which is the number of segments (de Waele and Broersen, 2000).

(4) $$AIC_{s}\left(p\right)=\text{ln}\left(RES\left(p\right)\right)+\frac{2p}{NS}$$

In this study, additional steps were taken to avoid overfitting. The Burg algorithm is prone to overfitting because it uses the same data to select the model order as are used to develop the model. Thus, in this study a portion of the data set was held out from the model development and used to select the final model. In this process, the final model was selected based on another metric, the Kullback-Leibler discrepancy (KLD). The KLD is a generalized error measure for two probabilistic distributions, *p*(*x*) and *q*(*x*) (Kullback, 1959).

(5) $$D\left(p∥q\right)=\int_{x}p\left(x\right)\text{log}\frac{p\left(x\right)}{q\left(x\right)}$$

In this case, *p*(*x*) represents the probabilistic distribution estimate of the model from the Burg algorithm, and *q*(*x*) the probabilistic distribution of the held-out set. In fact, the AIC is an estimate of the KLD that is specialized for measuring the distance between a set of realizations of a random process and a model developed based on them (Burnham and Anderson, 2004; De Waele, 2003). However, in general, the AIC may not be appropriate for estimating the distance from a model to another independent set (De Waele, 2003); thus, the KLD was adopted for selecting the final model using the held-out set.

## Materials and Methods

MALDI TOF mass spectra were measured from a blank plate to obtain noise from instrumentation. This type of noise is generated by electric circuits (e.g. the transimpedance amplifier, power supply and power line, and the ion accelerator pulse) in the instrument and electric/magnetic interferences from nearby equipment. Since no actual ion particle detection is performed in the experiments, noise from instrumentation does not include the noise caused by the ion detector. Since the gold coating of the plate can cause chemical noise if the laser hits it, we ensured that the laser was not directly illuminating the plate by installing a physical barrier between them. A total of six data sets were created using three MALDI TOF machines of two types to investigate how the power spectral density of noise from instrumentation varies with machine type, location, and time. Table 2 summarizes the data sets that were used in our study. Data were collected on October 7th, 2005 and October 17th, 2005 using two Voyager Biospectrometry instruments (Applied Biosystems, Framingham, MA, USA) located in two separate proteomics core facilities of the University of Texas at Austin (UT Austin). The acceleration voltage of the mass analyzer was set to 28,125 V. Each spectrum was the average of 256 individual scans and had 262,144 data points with a bin size of 10 ns (sampling rate). Each UT data set consisted of 20 mass spectra. Averaging multiple scans to obtain a mass spectrum has been traditionally accepted to reduce the randomness that may occur in data acquisition, which can be considered as an elementary noise reduction scheme. Therefore, we also investigated the potential effects of noise from instrumentation on mass spectra by deriving an AR model based on the average of individual scans. It should be noted that the average of individual scans is still a random process, so certain statistics like the PSD can be derived from it. Data were also collected on November 4th, 2005 and November 21st, 2005 using a third machine, a Voyager STR MALDI TOF instrument (Applied Biosystems, Framingham, MA, USA), located at the Moffitt Cancer Center (MCC). The acceleration voltage of the mass analyzer was set to 25,000 V. Each mass spectrum was the average of 250 scans and had 233,889 data points with a bin size of 10 ns. Each MCC data set consisted of 20 mass spectra. In each data set, 10 mass spectra were randomly selected and held out as a validation group to determine the optimal model order and the remaining 10 mass spectra were used to develop a linear model for noise from instrumentation. Some summary statistics of the MALDI TOF mass spectra are presented in Table 3. The mean DC offset was estimated by taking the mean of the means of individual mass spectra belonging to the same set (Equation (6)). Similary, the mean root-mean-square (RMS) amplitude was calculated after centering each mass spectrum at zero (i.e. subtracting the mean from each mass spectrum) using Equation (7). In Equation (6) and (7), *x_m_*(*n*) represents the *n*th point of the *m*th realization of noise from instrumentation.

(6) $$x^{DC}=\frac{1}{MN}\sum_{m=1}^{m=M}\sum_{n=1}^{n=N}x_{m}\left(n\right)$$

(7) $$x^{RMS}=\frac{1}{M}\sum_{m=1}^{M}\left(\sqrt{\frac{1}{N}\sum_{n=1}^{N}{\left(x_{m}\left(n\right)-\frac{1}{N}\sum_{n=1}^{N}x_{m}\left(n\right)\right)}^{2}}\right)$$

The Burg algorithm for multiple segments was applied to the training portion of each of the six data sets to obtain an AR model for noise from instrumentation for each of the machines. Because the DC offset of mass spectra introduces bias in the model parameters, the DC offset must be estimated and subtracted (de Waele and Broersen, 2000). In our study, the means of individual mass spectra were used as the estimate of the DC offset. The Burg algorithm for segments was implemented by de Waele and Broersen (de Waele and Broersen, 2000) using MATLAB^®^ (TheMathworks, Natick, MA, USA), and their toolbox is publicly available (http://www.mathworks.com). This MATLAB^®^ implementation allows the user to limit the maximum model order to control the complexity of the model. The Burg algorithm for segments was used to develop a model on the training portion of the data. The algorithm uses AIC to select the optimal model order, on the training data, up to the specified maximum model order. The entire process was repeated several times with the maximal model order parameter varying from 100 to 10,000. The final model was selected from among this set of possible models using the validation set. The average KLD between each model and the held-out mass spectra was calculated and the model with the smallest average KLD was selected as the optimal model for the data set.

Once the final models for the data sets were determined, the power spectral densities of the models were obtained using a Fourier transform from the model parameters. A sharp peak of the power spectral density at a certain frequency means that a strong sine wave with the frequency exists in the noise. However, in order to fully understand how noise from instrumentation affects mass spectra, a true signal without noise (e.g. mass spectrum free from noise) would also be needed. Since this cannot be obtained in general, a simulation was performed in our study in order to reveal the effect of only noise from instrumentation on MALDI TOF mass spectra

The potential effect of noise from instrumentation was investigated by adding simulated noise to simulated noise-free MALDI TOF mass spectra. Noise from instrumentation was simulated based on data generation methods proposed by Broersen and de Waele (Broersen and de Waele, 2003), which can generate a random process given an AR model obtained from the Burg algorithm. Because the noise generator produces a standard stationary random signal with zero-mean and unit-standard deviation, the simulated noise was compensated to have the mean and standard deviation estimated from real mass spectra of noise from instrumentation. Noise-free MALDI TOF mass spectra were simulated using the MALDI TOF simulation model developed by Coombes et al. (Coombes et al. 2004), which we translated from S-PLUS^®^ to MATLAB^®^. Coombes et al’s MALDI TOF model includes several key aspects of the MALDI TOF process such as peak broadening due to the distribution of isotopes and initial ion velocities. Generally, 100s–1,000s molecules are ionized per laser shot with initial velocities whose mean and standard deviation are 350 m/s and 50 m/s respectively during the MALDI TOF process (Beavis and B.T., 1991, Juhasz et al. 1997). In our simulation, it was assumed that 1,000 molecules (≈1.7 × 10^−21^ moles) are ionized in each laser shot. Microchannel plate (MCP) detectors, commonly used in MALDI TOF, amplify the signal for detected ions by a factor of 10^2^–10^4^ (Koppenaal et al. 2005). Generally, TOF mass spectrometers employ the chevron MCP as a detector, which provides a gain of about 10^6^–10^7^ per ion collision (Ladislas Wiza, 1979). Since the specifications of the transimpedance amplifier after the detector is not publicly available, our simulation assumes a total gain of 10^7^ in ion detection and that the MCP generates no additional noise (e.g. shot noise in the detector). A total of 57 proteins contained in human plasma were simulated. The number of proteins molecules ionized by the MALDI process was calculated based on the relative concentration ratios of these proteins in human plasma (Anderson and Anderson, 2002). Each simulated mass spectrum was assumed to be externally calibrated using six calibrants (m/z = 175.2, 1060, 5734, 12360.5, 16951.5, 66430: arginine, bradykinin, bovine insulin, cytochrome C, myoglobin, bovine serum albumin) using the least square error method.

## Results

**Figure 1.**
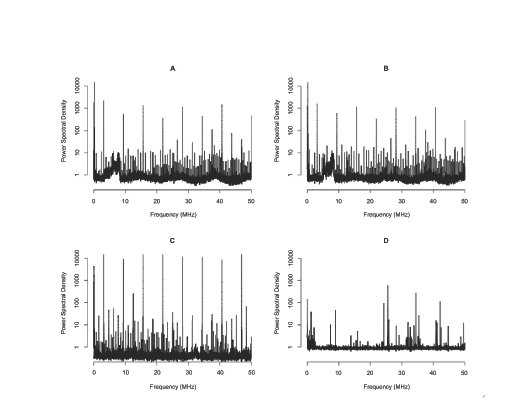
Power spectral densities of the AR models obtained from (**A**) SetA_UT1, (**B**) SetB_UT1, (**C**) SetA_UT2, and (**D**) SetA_MCC. When comparing (**A**) and (**B**), the frequency characteristics of noise from instrumentation in the same MALDI TOF instrument do not vary over dates of collection. Two MALDI TOF instrument of the older model type (Voyager Biospectrometry) show similar power spectral densities ((**B**) and (**C**)) containing prominent harmonics and more periodic components. In comparison, the instrument of the newer model type (Voyager STR) shows no noticeable harmonics and fewer periodic components in its power spectral density (**D**).

In a plot of the power spectral density, the x-axis represents the frequency (linear scale) and the y-axis represents the normalized power of each periodic component in noise (logarithmic scale). In general, a mass spectrum shows the relative abundances of protein/peptide species given in a sample, which are actually the digitized values of the output voltage from the transimpedance amplifier connected to the ion detector; however, since the unit of those values is not clearly provided by the manufacturer, the unit of power spectral density was not specified in this paper. The power spectral density was normalized with respect to the power gain between the input, in this case a white Gaussian random signal with an unit variance, and the output of the linear signal model established by the Burg algorithm for segments. The power spectral densities for spectra collected on the same machine on different days are similar (e.g. compare Fig. 1 A and B). Thus, the power spectral density of noise from instrumentation remains stable over the time scale of this study, which shows that noise from instrumentation can be modeled as a stationary random process.

**Figure 2.**
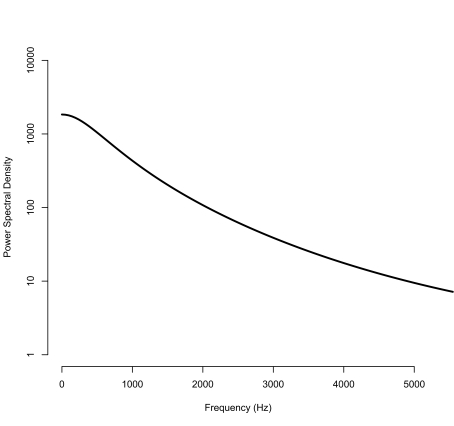
The zoomed-in power spectral density of SetA_UT1 (0–5,000 Hz). The power spectral density clearly shows 1/f noise in the low frequency region. The DC offset is not zero even though the means of the individual mass spectra of noise from instrumentation were removed before the Burg algorithm for segments was applied. The DC offset may be explained by the bias between the true mean and the mean estimates of the mass spectra and the effect of 1/f noise near 0 Hz.

Below 10 kHz, it was observed that the noise power at 0 Hz is non-zero and monotonically decreases until about 5 kHz in all of the power spectral density estimates (Fig. 2). In the higher frequency region, many peaks are observed in the power spectral densities of the data from UT, which indicates that mass spectra from those instruments may be affected by electric or magnetic interferences in addition to thermal noise (Fig. 1 A and C). Harmonics that begin at 3.125 MHz and continue at an interval of 6.25 MHz until 40.625 MHz are present in the power spectral densities of the UT instruments, which are identical models located in separate facilities. The MALDI TOF instruments of the same model (UT1 and UT2) showed very similar power spectral densities except for the peaks from 5–10 MHz, which are believed to be environmental interferences peculiar to the UT1 instrument. The power spectral densities of different models of MALDI TOF instruments were also obtained and compared (Fig. 1 D). Unlike the power spectral densities of the UT instruments, the power spectral densities of the spectra from the MCC machine do not have regular patterns like harmonics. Moreover, fewer periodic components were observed in the MCC power spectral density than in those of the instruments at UT Austin (compare Fig. 1 A and D).

**Figure 3.**
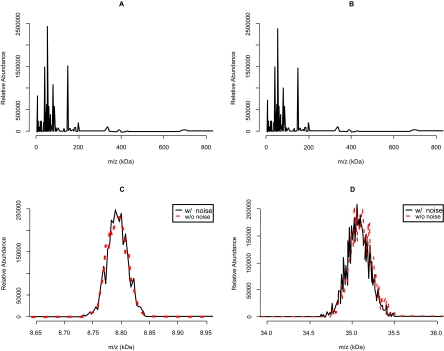
Simulated human plasma mass spectra. It is assumed that about 1,000 molecules are ionized every laser illumination, and the gain of the ion detector is 10^7^. (**A**) The entire view of the mass spectrum without noise from instrumentation. (**B**) The entire view of the mass spectrum with noise from instrumentation. (**C**) A zoomed view of a MALDI mass spectrum showing a peak near m/z 8.8 kDa. (**D**) A zoomed view of mass spectrum near 35 kDa. In (**C**), and (**D**), the black solid lines represent mass spectra with noise, and the red dashed lines mass spectra without noise. In (**D**), the peak with noise from instrumentation is not clearly distinguished from that without noise from instrumenta-

The power spectral density of noise is extremely useful when designing digital filters because the power spectral density informs which periodic components are dominant in signal deterioration, and thus should be removed. However, it is difficult to determine how noise from instrumentation affects mass spectra by looking at only its power spectral density. Thus, the impact of noise from instrumentation was investigated by adding noise simulated based on the noise model to simulated noise-free MALDI TOF mass spectra. Figure 3 A and B present the full view of the simulated mass spectrum without any types of noise and the one corrupted by noise from instrumentation simulated based on the SetA_UT1 power spectral density estimates. These noise-free and noisy mass spectra look almost identical at glance. Figure 3 C and D shows the zoomed-in views of peaks at 8,800 and 35,000 *m*/*z*, respectively, to further investigate the effects of noise from instrumentation. As these figures demonstrate, no significant differences are visually apparent between the noise-free and noisy mass spectra. From the simulation results, we note that noise from instrumentation does not appear to make a significant impact on the quality of MALDI TOF mass spectra.

## Discussion and Conclusion

The power spectral density reveals how the power of the periodic components hidden in the noise is distributed with respect to frequencies given a random process, and thus helps in developing filtering strategies for noise reduction. In our study, noise from instrumentation was separated from other types noise in MALDI TOF MS, and its power spectral density was estimated using the Burg algorithm for multiple segments, which develops an AR model for the noise by minimizing the residuals between the model and multiple observed noise segments simultaneously. The Burg method for segments provides much less biased models than other methods such as parameter averaging methods when multiple signal segments from the same source are available for parameter estimation (de Waele and Broersen, 2000). Thus, this algorithm is well suited for the purpose of estimating the power spectral density of a random process with a finite length, but multiple realizations available like noise from instrumentation.

Interesting features of noise from instrumentation were observed in our studies. Since the DC offsets of the mass spectra were already removed individually before applying the Burg algorithm for segments, there should not be a peak at 0 Hz; however, as can be seen in Figure 2, our power spectral density estimate is not zero at 0 Hz. This power component at 0 Hz may have been caused by the bias between the estimated DC offset of mass spectra and the true value. The bias cannot be completely removed since only a finite number of noise realizations are available, affecting the model parameters that determine the DC power component in the power spectral density (de Waele and Broersen, 2000). Nonetheless, these high values near 0 Hz may not be completely explained by the bias in the model parameters. One possible hypothesis is that this noise component may originate from 1/f noise of the MALDI TOF instrument (Fig. 2). This type of noise is also called “pink noise” or “flicker noise” and is known to be mainly due to a fluctuation of the mobility of the free charge carriers in an electronic device, and it is characterized by the inverse relationship between the frequency and the power spectrum (Ott, 1988). 1/f noise has a far narrower bandwidth than other types of noise such as white noise, mainly affecting low frequency signals. Therefore, we suspect that 1/f noise also contributes to the non-zero values near 0 Hz in the power spectral density.

To see the variation of the power spectral density with the instrument type, location, and date of collection, six data sets of noise from instrumentation were measured from three different MALDI TOF instruments. The power spectral density does not vary much over the time scale studied, but it varies with the instrument type and location. This observation is also consistent with our assumption that noise from instrumentation is a stationary random process, which is a critical requirement for applying power spectral density analysis. The power spectral density of a stationary random process is consistent across realizations of the process over time because the statistical characteristics (i.e. the ensemble mean and autocorrelation) of a stationary random process are time-invariant.

The comparison of the power spectral densities from two identical instruments located in the different facilities suggests that both internal and external electric or magnetic interference sources affect the mass spectra. More specifically, the fact that the same harmonics are observed in both devices at UT implies that the source of this interference is within the mass spectrometer (compare Fig. 1 A and C). On the other hand, there are non-harmonic periodic components present in the power spectral density for one of the UT instruments but not the other (compare Fig. 1 A and C). The absence of these periodic components in the UT2 power spectral density suggests that external sources generating electric or magnetic interference ranging from 5 MHz to 10 MHz may exist near the UT1 MALDI TOF instrument, but not near UT2 since these instruments are the same machine type, but located in different facilities. Therefore, shielding should be carefully considered to avoid signal deterioration due to the interference from nearby equipment. In principle, this hypothesis could be tested by systematically turning off all other instruments in the facility and re-analyzing the mass spectra of noise from instrumentation. However, it is not practical to turn off all nearby equipment because the MALDI TOF instruments are located in the proteomics core facilities, where many other research experiments are run simultaneously.

**Figure 4.**
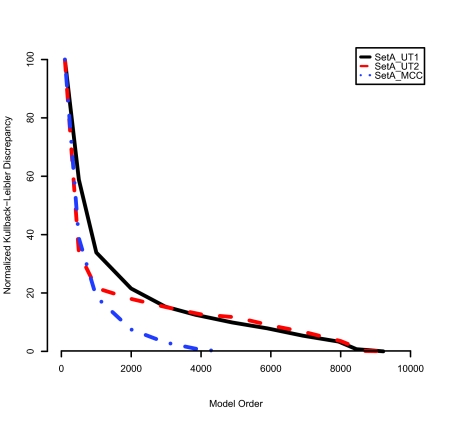
Normalized KLDs of the AR models with respect to the validation mass spectra. The KLD of each AR model is normalized with respect to its maximum and minimum values, and then multiplied by 100. The black solid line is the KLD of SetA_UT1, the red dashed line is that of SetA_UT2, and the blue dashed dot line is that of SetA_MCC. The optimal model order of each model is decided at the point where its KLD stops decreasing.

The power spectral density and model order optimization analyses imply that the newer MALDI TOF instrument (Voyager STR, Applied Biosystems, Framingham, MA, U.S.A.) at MCC may employ more effective noise shielding schemes than the earlier model. That is, fewer periodic components are seen in the power spectral densities of the newer instrument than in those of the older ones. This is probably a consequence of more advanced instrumentation design of the newer model that provides better shielding to the internal or external interference. The average KLD of AR models with respect to the validation mass spectra provides additional evidence (Fig. 4). For SetA_UT1, the average KLD decreases as the model order is increased up to about 9,000 and then plateaus; thus, the optimal model order is the maximum order of 9,000. Similarly, the optimal AR model order for SetA_UT2 is approximately 8,500. However, the KLD of the model for SetA_MCC plateaus at about 4,500, which suggests that the power spectral density of SetA_MCC may contain fewer periodic components than those of SetA_UT1 and SetA_UT2 since each term in the AR model represents a periodic component with a specific frequency.

The potential effect of noise from instrumentation was investigated through a simulation study. The simulation suggests that only noise from instrumentation may not significantly impact the interpretation of mass spectra. In fact, the RMS magnitude of noise is almost negligible in the high mass region when it compared to the randomness of the peak shapes due to ions’ random initial velocities (Fig. 3 D). This is consistent with the fact that the DC offset and root-mean-square (RMS) magnitudes of noise from instrumentation are relatively small, ranging only 250–1,500 and 6 to 11, respectively (Table 3), which are negligible compared to the height of the example peaks (≈250,000).

In conclusion, this paper presents a systemic methodology for modeling noise from instrumentation in MALDI TOF MS on the basis of parametric power spectral density estimation using multiple realizations. Our study opens a way of isolating a noise component, and measuring its stochastic features, which are critical in designing filters for signal manipulation often needed for MS applications like biomarker identification. In addition, this methodology will also benefit system designers of mass spectrometers as well by providing reliable spectral information on noise, letting them developing better shielding strategies for potential signal interference. For example, in our study, the power spectral densities of the mass spectrometers of the earlier model indicate that more shielding should be considered to avoid the periodic interference for a higher signal quality although the overall impact of noise from instrumentation was assessed to be low according to our simulation study. In future studies, similar approaches could be applied to other types of noise in MALDI TOF MS such as chemical noise. Isolating individual subtypes of noise and performing stochastic modeling of them will provide an important perspective on how to suppress signal deterioration due to the noise effectively by showing the power distribution over frequencies. Furthermore, such noise analysis can also be extended to other types of instrumentation like ESI MS once the types of noise in the instrumentation are identified and isolated. Hence, this technique is expected to benefit noise reduction studies for other types of MS instrumentation as well.

## Figures and Tables

**Table 1.** The comparison of non-parametric and parametric power spectral density estimation. Under the situation that only realizations of noise from instrumentation with a finite length are available, parametric power spectral density estimation has more advantages over non-parametric estimation while longer computation time is needed.

**Method**	**Advantage**	**Disadvantage**
Non-parametric	Simple Easy to use	Poor frequency resolution Spectral leakage

Parametric	Unlimited frequency resolution No spectral leakage Easy to use for simulation	Relatively complex modeling process

**Table 2.** The data sets of noise from instrumentation. The data sets were measured to investigate how the power spectral densities of noise from instrumentation varied with instrument type, time and location.

**Data set**	**Location**	**Date**	**Number of MS**
SetA_UT1	UT Austin, Mass Spectrometry and Proteomics Facility	10/07/2005	20
SetB_UT1		10/17/2005	20

SetA_UT2	UT Austin, Institute of Cell & Molecular Biology Core Facility	10/07/2005	20
SetB_UT2		10/17/2005	20

SetA_MCC	H. Lee Moffitt Cancer Center	11/04/2005	20
SetB_MCC		11/21/2005	20

**Table 3.** The average DC offset and average RMS magnitude of mass spectra in each data set in relative intensity. As can be seen in this table, these statistics are consistent over time, but vary across the instruments. The potential effect of noise from instrumentation was investigated by adding simulated noise to simulated noise-free MALDI TOF mass spectra. These DC offsets and RMS magnitudes are needed in generating simulation noise using the models obtained from our parametric power spectral density analysis.

**Data set**	**Average DC offset**	**Average RMS magnitude**
SetA_UT1	255.0	6.9
SetB_UT1	281.4	6,8
SetA_UT2	844.5	11.9
SetB_UT2	905.9	11.2
SetA_MCC	1425.4	6.8
SetB_MCC	1523.5	5.4
